# Spread of Measles Virus D4-Hamburg, Europe, 2008–2011

**DOI:** 10.3201/eid1708.101994

**Published:** 2011-08

**Authors:** Annette Mankertz, Zefira Mihneva, Hermann Gold, Sigrid Baumgarte, Armin Baillot, Rudolph Helble, Hedwig Roggendorf, Golubinka Bosevska, Jasminka Nedeljkovic, Agata Makowka, Veronik Hutse, Heidemarie Holzmann, Stefan W. Aberle, Samuel Cordey, Gheorghe Necula, Andreas Mentis, Gulay Korukluoğlu, Michael Carr, Kevin E. Brown, Judith M. Hübschen, Claude P. Muller, Mick N. Mulders, Sabine Santibanez

**Affiliations:** Author affiliations: Robert Koch Institute, Berlin, Germany (A. Mankertz, S. Santibanez);; National Center of Infectious and Parasitic Diseases, Sofia, Bulgaria (Z. Mihneva);; Department of Health and Environment, München, Germany (H. Gold);; Institute of Hygiene and the Environment, Hamburg, Germany (S. Baumgarte);; Governmental Institute of Public Health of Lower Saxony, Hannover, Germany (A. Baillot);; Health Office City of Mannheim, Mannheim, Germany (R. Helble); Public Health Office, Essen, Germany (H. Roggendorf);; Institute for Public Health, Skopje, Republic of Macedonia (G. Bosevska);; Institute of Virology, Belgrade, Serbia (J. Nedeljkovic); National Institute of Public Health, Warsaw, Poland (A. Makowka);; Scientific Institute of Public Health, Brussels, Belgium (V. Hutse);; Medical University of Vienna, Vienna, Austria (H. Holzmann, S.W. Aberle);; University of Geneva Hospitals, Geneva, Switzerland (S. Cordey);; Cantacuzino National Institute of Research and Development for Microbiology and Immunology, Bucharest, Romania (G. Necula);; Hellenic Pasteur Institute, Athens, Greece (A. Mentis); Refik Saydam National Public Health Agency, Ankara, Turkey (G. Korukluoğlu);; Virus Reference Laboratory, Dublin, Ireland (M. Carr); Health Protection Agency, London, UK (K.E. Brown);; Laboratoire National de Santé/CRP-Santé, Luxembourg (J.M. Hübschen, C.P. Muller);; World Health Organization Regional Office for Europe, Copenhagen, Denmark (M.N. Mulders)

**Keywords:** measles, D4-Hamburg, outbreak, elimination, vaccination, transmission, genotyping, Roma, ethnic minority group, viruses, Europe, research

## Abstract

A new strain of measles virus, D4-Hamburg, was imported from London to Hamburg in December 2008 and subsequently spread to Bulgaria, where an outbreak of >24,300 cases was observed. We analyzed spread of the virus to demonstrate the importance of addressing hard-to-reach communities within the World Health Organization European Region regarding access to medical care and vaccination campaigns. The D4-Hamburg strain appeared during 2009–2011 in Poland, Ireland, Northern Ireland, Austria, Greece, Romania, Turkey, Macedonia, Serbia, Switzerland, and Belgium and was repeatedly reimported to Germany. The strain was present in Europe for >27 months and led to >25,000 cases in 12 countries. Spread of the virus was prevalently but not exclusively associated with travel by persons in the Roma ethnic group; because this travel extends beyond the borders of any European country, measures to prevent the spread of measles should be implemented by the region as a whole.

## Abstract 2

TOC Summary: More than 24,300 cases were identified in 22 countries.

The 53 member states of the World Health Organization (WHO) European Region (EUR) have set a goal to eliminate measles and rubella virus transmission by 2015 in Europe (*1*). Elimination targets include 95% vaccination coverage with 2 doses of measles virus–containing vaccine (MVCV), an incidence of <1 measles case per million population, 80% of outbreaks associated with <10 cases, and transmission of indigenous or imported measles virus for no longer than 12 months in a defined region (*2*). Thus, monitoring transmission chains of measles virus is an indispensable tool to assess elimination progress, although the specific boundaries of the region have not yet been defined for the WHO EUR.

To comply with the goal of eliminating measles virus, Germany implemented a national intervention program against measles, mumps, and rubella (MMR) in 1999 (*3*). Since then, measles incidence in Germany has declined. Molecular surveillance showed that endemic genotypes C2 (MVi/Kempten.DEU/23.00) and D6 (MVi/Berlin.DEU/47.00) (*4*) were replaced rapidly by genotype D7 (MVi/Mainz.DEU/06.00), which circulated until the beginning of 2003 (*5*). Imported measles virus of genotypes B3, D4, D5, D6, D8, D9, and H1 appeared in Germany from 2005 onward. In 2009 and 2010, most cases were linked to measles virus of genotype D4, of which several distinct subvariants were detected.

Elimination targets have not yet been met in Germany. Vaccination coverage in Germany, routinely assessed in children 5–6 years of age during an examination before school entry, is still below the required 95% for the second dose of MVCV. Recent outbreaks showed an immunization gap in adolescents and young adults (*6*). Consequently, outbreaks still occur in Germany every year, although recently they have been more limited in number of cases, length of time, and extent of national transmission (*7**,**8*). A total of 915 measles cases were reported in 2008, 571 in 2009, and 780 in 2010; incidence was 7–10 cases/1 million population (www3.rki.de/SurvStat/QueryFormaspx).

The reasons for Germany’s malperformance are complex. Measles virus vaccination is not mandatory, and some groups within the German population do not comply with official vaccination recommendations (*9*) because of philosophical or religious beliefs or fear of adverse effects (*10*). As in other countries in Europe, strategies to address hard-to-reach populations and improve access to medical care, preventive measures, and vaccination campaigns have not yet been developed. In this article, we describe exportation of a measles D4 variant from Germany and its subsequent circulation in Europe.

## Material and Methods

Serum, urine, and oral fluid or throat swabs were sent to the WHO Regional Reference Laboratory in Berlin according to the procedures outlined in the WHO LabNet manual (*11*). Immunoglobulin (Ig) M and IgG serologic testing was performed as described previously (*12*). Sequencing was performed according to WHO recommendations (*13*). Sequences were aligned by ClustalW (*14*) and further analyzed by SeqScape 2.5 and MEGA4 DNA analysis software (*15*). Phylogenetic trees were constructed by using the neighbor-joining method. Genotype assignment was performed by phylogenetic comparison with the measles virus reference strains as designated by WHO (*16*). The obtained sequence data, the genotype, the official WHO measles virus sequence name, and relevant epidemiologic data were submitted to the WHO measles sequence data­base, MeaNS (www.hpa-bioinformatics.org.uk/Measles/Public/Web_Front/main.php) or to GenBank.

## Results

### Outbreak Hamburg/Lower Saxony

At the end of December 2008, a 27-year-old man residing in the southern part of Hamburg was hospitalized with measles. Five other adults contracted the virus while waiting for treatment in the emergency room. The resultant outbreak first affected a southern quarter (Hamburg-Harburg) of the city of Hamburg. It spread subsequently into a Roma group residing in central Hamburg and to the neighboring federal state of Lower Saxony. The probable index case-patient was identified as a 19-year-old Roma man living in Hamburg. He had stayed in London from May through November 2008 and became ill at the beginning of December with measles-like symptoms. The outbreak in Hamburg lasted from the end of December 2008 through June 2009 (*17*); a 4-week peak occurred in February and comprised 216 cases in the city of Hamburg.

For 69% of the reported cases, the diagnosis was laboratory confirmed. Complications leading to hospitalization were seen in 40% of the patients (pneumonia or otitis media). The affected age group ranged from a 1-day-old newborn to a 54-year-old adult (median age 13.5 years). Most frequently affected were young children, then young adults. Data with respect to vaccination status were available for 196/216 case-patients. No vaccination was documented for 167 persons (85%); 28 had not yet reached the age of vaccination (>11 months). Twenty-six (13%) previously unvaccinated persons had received MVCV after being exposed to measles virus. Three patients had received 2 doses of MVCV. Several of the 216 cases occurred in the Roma ethnic community.

Seventy-two cases of measles were reported during the same time in Lower Saxony. Fifty-three cases were clearly related to the outbreak in Hamburg. The first cases in Lower Saxony were reported during week 2 (January) and the last case occurred during week 17 (April) of 2009; the peak of the outbreak occurred during week 14 (April). The connection to the Hamburg outbreak was suggested either by the presence of patients in the emergency department of a Hamburg hospital at the time in question, an epidemiologic link, or the result of the sequencing. Many cases occurred in the Roma ethnic group.

Case-patients ranged from 7 months to 42 years of age (median age 15 years); adolescents and younger adults were the main affected age group. Forty-two (79%) case-patients had received no measles vaccination, 10 (19%) had received 1 dose of MVCV, and 1 (2%) had been vaccinated 2 times. In the latter case, primary infection with measles virus was confirmed by PCR and IgM, but IgG was not detected. Five patients received vaccination after exposure, which did not prevent clinical symptoms. Overall, 47 (89%) of measles cases were confirmed by laboratory testing. Eleven (21%) case-patients were admitted to a hospital with complications (e.g., pneumonia, otitis media).

**Figure 1:**
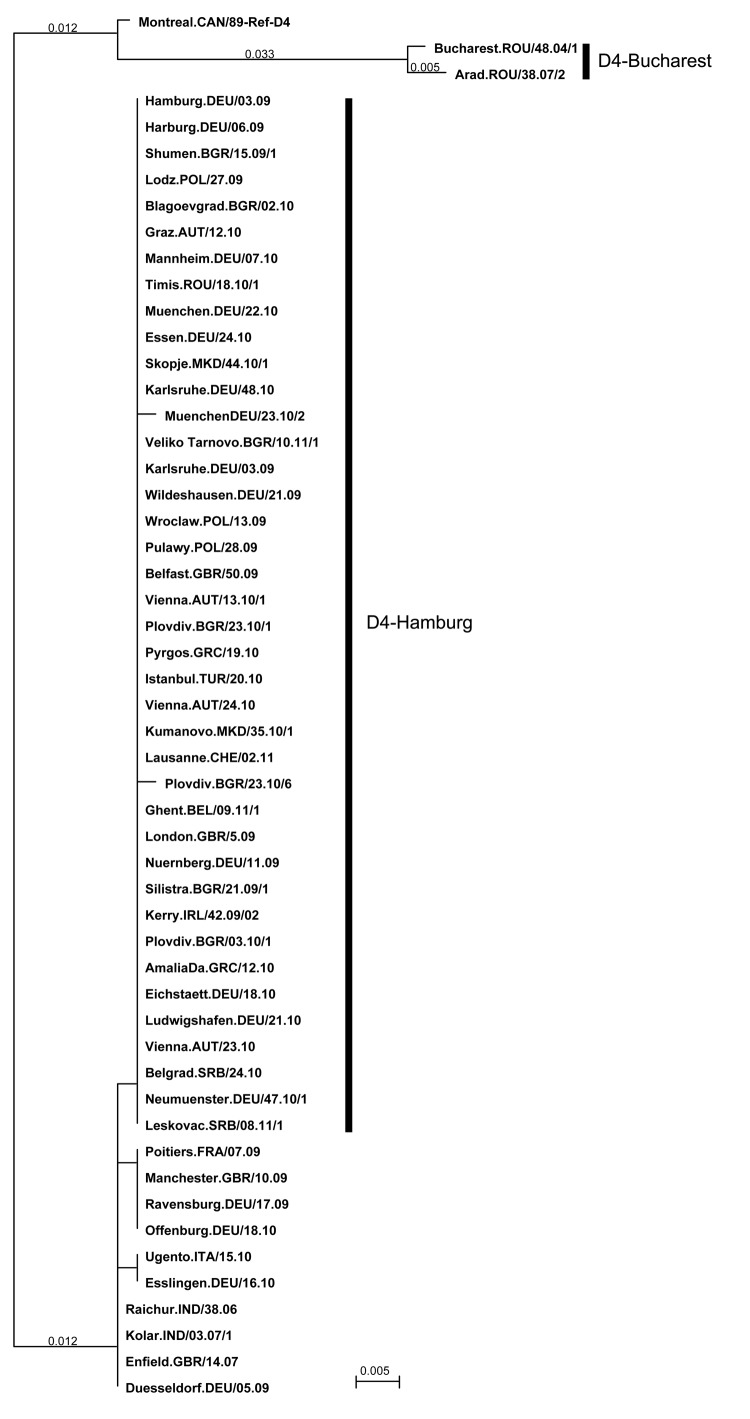
Phylogenetic relationships between measles viruses of genotype D4 recently detected in Europe. The measles virus variant D4-Hamburg initiated a long-lasting transmission chain spreading to several European countries during 2008–2011. D4-Hamburg belongs to the D4-Enfield lineage, which is genetically distinct from the previously widespread lineage D4-Bucharest. Phylogenetic analysis is based on a 456-nt sequence encoding the C-terminus of the measles virus nucleocapsid protein. The tree was constructed by the neighbor-joining method by using MacVector version 11.1.2 software (www.macvector.com). Scale bar indicates number of 5-nt deviations per 1,000-nt sequence. GenBank accession numbers are MVi/Montreal.CAN/89 (World Health Organization reference strain; www.cdc.gov/measles/lab-tools/who-table.html), U01976; MVs/Bucharest.ROU/48.04/1, AM849091; MVs/Arad.ROU/38.07/2, HQ704309; MVs/Hamburg.DEU/03.09, HQ436108; MVs/Wildeshausen.DEU/21.09, HQ704360; MVs/Nuernberg.DEU/11.09, HQ436110; MVs/Lodz.POL/27.09, HQ441202; MVs/Belfast.GBR/50.09, GU479875; MVs/Vienna.AUT/13.10/1, HQ704298; MVs/Plovdiv.BGR/23.10/1, HQ436106; MVs/Pyrgos.GRC/19.10, HM802126; MVs/Istanbul.TUR/20.10, HM579947; MVs/Vienna.AUT/24.10, HQ704300; MVs/Kumanovo.MKD/35.10/1, not available (NA); MVs/Lausanne.CHE/02.11, pending; MVs/Plovdiv.BGR/23.10/6, HQ436107; MVs/Ghent.BEL/09.11/1, NA; MVs/Harburg.DEU/06.09, HQ436109; MVs/Wroclaw.POL/13.09, HQ441201; MVs/Silistra.BGR/21.09/1, HQ436104; MVs/Blagoevgrad.BGR/02.10, HQ704345; MVs/Graz.AUT/12.10, HQ441211; MVs/Mannheim.DEU/07.10, HQ704362; MVs/Kerry.IRL/40.09, NA; MVs/Timis.ROU/18.10/1, HQ704313; MVs/Muenchen.DEU/22.10, HQ704350; MVs/Essen.DEU/24.10, HQ704373; MVs/Skopje.MKD/44.10/1, NA; MVs/Karlsruhe.DEU/48.10, pending; MVs/MuenchenDEU/23.10/2, HQ704375; MVs/VelikoTarnovo.BGR/10.11/1, JF754464; MVs/Karlsruhe.DEU/03.09, HQ436113; MVs/London.GBR/5.09, GU120179; MVs/Shumen.BGR/15.09/1, HQ436103; MVs/Pulawy.POL/28.09, HQ441203; MVs/Plovdiv.BGR/03.10/1, HQ436105; MVs/AmaliaDa.GRC/12.10, HM802121; MVs/Eichstaett.DEU/18.10, HQ704346; MVs/Ludwigshafen.DEU/21.10, HQ704349; MVs/Vienna.AUT/23.10, HQ704299; MVs/Belgrad.SRB/24.10, NA; MVs/Neumuenster.DEU/47.10/1, JF754463; MVs/Leskovac.SRB/08.11/1, NA; MVs/Poitiers.FRA/07.09, FN663615; MVs/Manchester.GBR/10.09, GQ370461; MVs/Ravensburg.DEU/17.09, HQ436112; MVs/Offenburg.DEU/18.10, HQ704368; MVs/Ugento.ITA/15.10, HM173092; MVs/Esslingen.DEU/16.10, HQ704364; MVs/Raichur.IND/38.06, EU812270; MVi/Kolar.IND/03.07/1, EU812284; MVs/Enfield.GBR/14.07, EF600554; and MVs/Duesseldorf.DEU/05.09, HQ436111.

Specimens from 12 cases in Hamburg and 18 cases in Lower Saxony were genotyped. All case-patients were infected with the same D4 measles virus variant (MVs/Hamburg.DEU/03.09/[D4], MVs/Harburg.DEU/06.09/[D4], and MVs/Wildeshausen.DEU/21.09/[D4]), none of which were published in the GenBank and MeaNS databases. D4-Hamburg showed 1 mismatch to other D4 measles virus sequences published in GenBank, MVs/Raichur.IND/38.06/[D4]), MVi/Kolar.IND/03.07/1[D4], and MVs/Enfield.GBR/14.07/[D4]; the latter is a strain endemic to the United Kingdom and responsible for the large outbreak there during 2007–2009 (Figure 1). Sequences identical to the Hamburg strain were subsequently identified in London (MVs/London.GBR/5.09/[D4]).

### Transmission to Bulgaria

In April 2009, after an absence of 7 years, measles cases began occurring in Bulgaria (*18*). Sequencing of 3 specimens from the National Measles Laboratory in Sofia identified MVs/Shumen.BGR/15.09/1-3(D4), identical to D4-Hamburg. The index case-patient in the Bulgaria outbreak was a Roma who worked as a builder in Hamburg and who had visited Razgrad district in northeastern Bulgaria. The outbreak in Bulgaria proceeded from the northeast to the southwest of the country; in 2009, a total of 2,249 cases were reported. A marked increase in case numbers was reported at the end of 2009 and in the beginning of 2010 (*19*). From the start of the measles epidemic in April 2009 through the end of week 10 (mid-March) of 2011, a total of 24,379 cases were reported; 24 were fatal (*20*).

The WHO Regional Reference Laboratory in Berlin received 20 specimens at regular intervals from hospitalized persons. Genotype information was obtained for 19/20 case-patients (Table A1). All viruses detected showed the same sequence (MVs/Shumen.BGR/15.09/1-3[D4], MVs/Silistra.BGR/21.09/1-4[D4], MVs/Blagoevgrad.BGR/02.10[D4], MVs/Plovdiv.BGR/03.10/1-3[D4], MVs/Plovdiv.BGR/23.10/1-5[D4]), and MVs/VelikoTarnovo.BGR/10.11/1-2[D4]), with the exception of MVs/Plovdiv.BGR/23.10/6[D4], characterized by 1 mismatch (Figure 1).

### Laboratory Investigation of Measles Virus Samples from Bulgaria

Measles virus infection was reconfirmed for all 20 case-patients by positive test results for IgM, PCR, or both (Table A1). Results were correlated with the clinical data for each case-patient that had been compiled during hospitalization. For 12 case-patients, vaccination status was unknown; a 7-month-old baby was unvaccinated. Seven case-patients (1, 7, 12, 14, 15, 17, and 18) presented vaccination cards that stated the date of 1 or 2 vaccinations with MVCV (Table A1). All had positive IgM and PCR results; 2 had measles virus–specific IgG (case-patients 7 and 14). IgG avidity testing showed low avidity and thus a vaccination failure for case-patient 7. The equivocal IgM and the mediocre avidity of IgG in patient 14 did not indicate a primary infection. In summary, lack of immunologic response despite documented vaccination was apparent in 6 of 7 case-patients.

### Transmission of D4-Hamburg Strain in Europe

**Figure 2:**
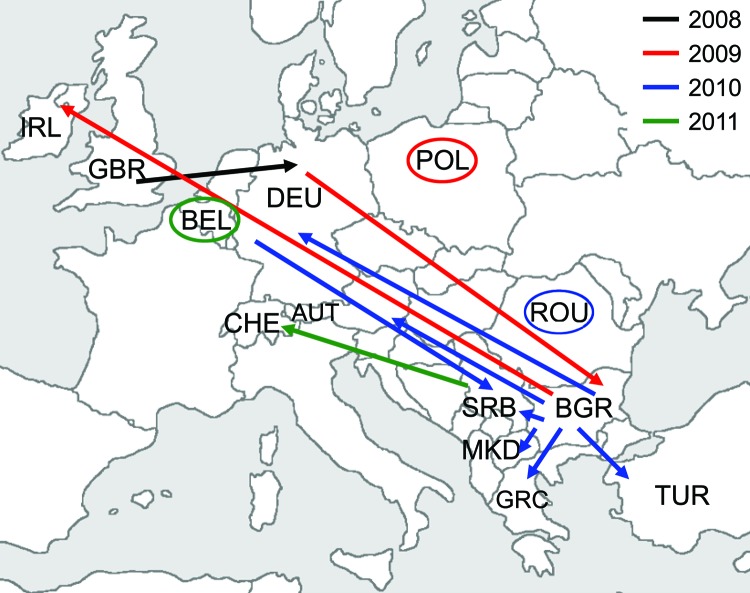
Transmission of the D4-Hamburg measles virus strain in Europe, 2008–2011. Arrows mark transmission with known epidemiologic link; ellipsoids mark detection without verified epidemiologic data. IRL, Ireland; GBR, Great Britain; BEL, Belgium; DEU, Germany; POL, Poland; CHE, Switzerland; AUT, Austria; ROU, Romania; SRB, Serbia; BGR, Bulgaria; MKD, Macedonia; GRC, Greece; TUR, Turkey.

WHO Regional Reference Laboratories in Berlin, Luxembourg, and London receive either specimens or sequence information from the national measles laboratories of 41 European countries. Sequencing of the 450-nt fragment of the N gene showed that the D4-Hamburg strain had further spread in Europe (Figure 2). Samples taken in Poland during the summer of 2009 showed infection with a virus identical to D4-Hamburg (Figure 1); a total of 54 cases were recorded during 2009, the first in June and the last in October. All were linked to 3 outbreaks among Roma residents in the towns of Lodz, Pulawy, and Olpole Lubelskie (MVs/Lodz.POL/27.09[D4], MVs/Pulawy.POL/28.09[D4]) (*21*). The virus was also exported to Ireland (MVs/Kerry.IRL/40.09[D4]) from the Roma population and from there into Northern Ireland (MVs/Belfast.GBR/50.09[D4]), with small clusters of associated cases in both countries.

In Austria, 4 cases classified as D4-Hamburg–associated were detected in March and June 2010. A first sporadic case occurred in Graz in March. A person of Bulgarian nationality who was a member of the Roma ethnic group was infected; he was staying in Austria at the time (MVs/Graz.AUT/12.10[D4]). Three additional cases belonged to a cluster observed among persons in Vienna who spoke Bulgarian (MVs/Vienna.AUT/13.10[D4], MVs/Vienna.AUT/23.10[D4], MVs/Vienna.AUT/24.10[D4]). D4-Hamburg was also seen in Greece, where the first cases and clusters at the beginning of 2010 were identified among families of Roma communities of Bulgarian nationality (MVs/Amaliada.GRC.12.10[D4], MVs/Pyrgos.GRC/19.10[D4]). The virus was then spread to persons of Greek nationality, mainly from Roma communities, reaching 91 laboratory-confirmed measles cases in 2010. Moreover, 2 sporadic cases of D4-Hamburg were observed in 2010 in Romania (MVs/Timis.ROU/18.10/1[D4]).

In Turkey, D4-Hamburg was detected in a tourist who stayed in Romania and Bulgaria before visiting Turkey (MVs/Istanbul.TUR/20.10/[D4]). In Serbia, D4-Hamburg was detected in a person with a sporadic case (MVs/Belgrad.SRB/24.10/[D4]) and in the Roma population during an outbreak in Leskovac (MVs/Leskovac.SRB/08.11/1[D4]); 13 persons were infected, of which 3 were hospitalized. The index case-patient was a person who returned at the end of November from Germany (Duisburg). Nearly 400 cases were detected in Macedonia (MVs/Kumanovo.MKD/35.10/1[D4], MVs/Skopje.MKD/44.10/1[D4]). Although we cannot be sure that D4-Hamburg is the only virus contributing to the current outbreaks in Serbia and Macedonia, ongoing transmission of D4-Hamburg is indicated by the recent detection of a sporadic case of D4-Hamburg in Switzerland (MVs/Lausanne.CHE/02.11[D4]; this person probably became infected in Serbia) and by an outbreak of >40 cases in Belgium (MVs/Ghent.BEL/09.11/1[D4]).

### Reimportation of the D4-Hamburg Strain to Germany

In 2010, D4-Hamburg measles virus was reimported to Germany. It appeared first in February in Mannheim, where specimens from 3 case-patients showed a sequence identical to D4-Hamburg (MVs/Mannheim.DEU/07.10[D4]). The virus was introduced by 8 persons from Bulgaria who belonged to a Turkish-speaking minority population, had acquired the infection in Dobrich (Bulgaria), and transmitted the virus to 3 relatives who were living in Mannheim. During June–August 2010, 48 measles cases were reported in Munich; 28 cases occurred among Bulgarian Roma residents in a migrant camp in eastern Munich. Several of these residents worked as cleaning staff at hotels in Munich. From these persons and other hospitalized members of the affected Roma group, the virus spread into the general population.

The age of case-patients in Munich ranged from 9 months to 36 years; 7 case-patients were <7 years of age, and 23 were >18 years of age. One case-patient was hospitalized because of encephalitis. Interviews with the help of an interpreter showed that none of the case-patients had MMR vaccination documents. Therefore, vaccination was offered to all inhabitants of the camp. Twenty-eight cases were investigated at the WHO Regional Reference Laboratory in Berlin. Twenty-three cases were associated with MVs/Muenchen.DEU/22.10[D4] identical with D4-Hamburg, and specimens from 5 members of the same group were closely related to MVs/Muenchen.DEU/23.10/2[D4]. Moreover, clusters and sporadic cases of D4-Hamburg were detected in several German cities, e.g., Eichstaett (2 cases, MVs/Eichstaett.DEU/18.10[D4]) and Ludwigshafen (1 case, MVs/Ludwigshafen.DEU/21.10[D4]) in May 2010. These cases were linked to importation of D4-Hamburg from Bulgaria and did not initiate virus spread within Germany. The same virus variant was also detected in a cluster of cases observed in the city of Essen during June–July 2010. This variant was imported from Bulgaria by a citizen of Bulgaria (MVs/Essen.DEU/24.10[D4]) and spread to another citizen of Bulgaria (MVs/Essen.DEU/25.10/1[D4]) and 6 persons of the general population (MVs/Essen.DEU/25.10/2[D4], MVs/Essen.DEU/28.10[D4]).

From week 47 (the end of November) on, 8 cases of infection with D4-Hamburg occurred in Neumuenster in northern Germany. This outbreak occurred in a home for migrants mainly from Afghanistan and Serbia (MVs/Neumuenster.DEU/47.10/1[D4]). From week 48 on, 6 cases were seen in another home for migrants in Karlsruhe (MVs/Karlsruhe.DEU/48.10[D4]).

## Discussion

A combination of epidemiologic data and genotyping results enabled us to trace the spread of measles virus D4-Hamburg in Europe. It was imported from London at the end of 2008 to northern Germany (288 cases), then transmitted from Hamburg to Bulgaria, where, after a 7-year absence of measles, an outbreak of 24,379 cases occurred. This was the largest outbreak seen in Europe since an outbreak in the Ukraine in 2006 (*22*).

Twenty cases from the outbreak in Bulgaria were sampled at different times (April and June 2009, January and June 2010, and March 2011) from persons in distinct districts. The samples were collected initially in northeastern and later in southwestern Bulgaria, thereby following the course of the outbreak. The cases were associated with measles virus sequences such as MVs/Shumen.BGR/15.09[D4], corresponding to D4-Hamburg. The only exception was MVs/Plovdiv.BGR/23.10/6[D4], which showed 1 mismatch but in all probability developed from MVs/Plovdiv.BGR/23.10/1–5[D4]. Because the samples had been obtained at different times and regions, our analysis provides substantial evidence that D4-Hamburg is responsible for the outbreak in Bulgaria, despite the small number of samples. Samples from 6 of 7 persons showed diagnostic markers of a primary measles infection, although these persons had a certificate of prior measles vaccination. Our results therefore demonstrate an urgent need to investigate the vaccination procedures for ethnic minorities.

D4-Hamburg was detected subsequently in Poland (54 cases) (*21*), Ireland, Northern Ireland, Austria (4 cases), Greece (149 cases) (*23*), Serbia (14 cases), Belgium (>40 cases), and Macedonia (>400 cases). Sporadic cases were detected in Romania, Turkey, and Switzerland. More than 70 D4-Hamburg–associated cases were detected in Germany after 8 separate reimportations. Taken together, D4-Hamburg was present in Europe from December 2008 to March 2011—that is, at least 27 full months—and caused >25,300 cases. Because sequencing results are not available quickly in most countries, this transmission chain is probably still ongoing. Circulation of imported measles virus for no longer than 12 months (and therefore endemic transmission according to the WHO definition) is a marker for successful elimination. We suggest, therefore, that the length of a given transmission chain should not be assessed on a national level but at the level of the all 53 countries within the WHO EUR.

Epidemiologic data showed that the spread of D4-Hamburg across Europe involved predominantly persons from the Roma ethnic group in Bulgaria. Another transmission chain affecting the Roma population in particular was recorded in 2004 in Romania. An outbreak of >8,000 cases associated with MVs/Bucharest.ROU/48.04[D4] commenced in the Roma population. Subsequent spread of D4-Bucharest by traveling Roma persons was observed until 2007 (*4*). The pronounced sequence deviation of D4-Bucharest and D4-Hamburg indicates the presence of at least 2 distinct and successive transmission chains in the Roma population. Both chains were long lasting and associated with a high number of cases, as well as several fatalities. This and other recent outbreaks in Roma communities (*7**,**24**,**25*) underline the need for the development of strategies to address this ethnic minority at the regional level and to improve their integration into the respective national health services.

The lack of strategies to address reaching the hard-to-reach communities in Europe will clearly have an adverse effect on the measles elimination process. In this context, we want to make clear that elimination of measles virus should not be seen exclusively as a Roma-associated problem. Measles virus is a highly infectious agent and will infect any population with low immunity rates. If itinerant groups are underserved by the national health sector, spread of measles virus is highly probable. Because measles outbreaks in western European countries occur mainly in undervaccinated groups (*26*), reaching the hard-to-reach is not the only important challenge. Thus, closing vaccination gaps in a setting of optional vaccination and vaccine skepticism is another important prerequisite that must be met on Europe’s path toward elimination of measles virus.

## Appendix

**Table A1:** Data for 20 patients and results of laboratory investigation of measles virus specimens, Bulgaria, April 2009–March 2011*

Patient no.	Age	Vaccination status, year	Rash onset/date specimens obtained	IgM	IgG	Avidity	PCR results	Genotype	Diagnosis
1	6 y	MMR1, 2004	2009 Apr 16/Apr 23	Pos	Equivocal	ND	U pos	MVs/Shumen.BGR/15.09/1[D4]	Acute measles, vaccination failure
2	29 y	Unknown	2009 Apr 16/Apr 23	Pos	Pos	ND	U pos	MVs/Shumen.BGR/15.09/2[D4]	Acute measles
3	33 y	Unknown	2009 Apr 16/Apr 23	Pos	Pos	ND	U pos	MVs/Shumen.BGR/15.09/3[D4]	Acute measles
4	13 y	Unknown	2009 May 25/May 30	Pos	Pos	ND	U pos	MVs/Silistra.BGR/21.09/1[D4]	Acute measles
5	23 y	Unknown	2009 May 25/May 30	Pos	Neg	ND	U pos	MVs/Silistra.BGR/21.09/2[D4]	Acute measles
6	11 y	Unknown	2009 May 25/May 30	Pos	Pos	ND	U pos	MVs/Silistra.BGR/21.09/3[D4]	Acute measles
7	11 y	MMR1, 1998	2009 May 25/May 30	Pos	Pos	Low (8%)	U pos	MVs/Silistra.BGR/21.09/4[D4]	Acute measles, vaccination failure
8	18 y	Unknown	2010 Jan 4/Jan 14	No serum	–	–	U pos	Sequence neg	Acute measles
9	11 y	Unknown	2010 Jan 12/Jan 21	Pos	Neg	ND	U pos	MVs/Blagoevgrad.BGR/02.10/1[D4]	Acute measles
10	22 y	Unknown	2010 Jan 14/Jan 21	Pos	Neg	ND	U pos	MVs/Plovdiv.BGR/03.10/1[D4]	Acute measles
11	7 mo	Not vaccinated	2010 Jan 18/Jan 21	No serum	–	–	U pos	MVs/Plovdiv.BGR/03.10/2[D4]	Acute measles
12	15 y	MMR2	2010 Jan 13/Jan 21	Pos	Neg	ND	U pos	MVs/Plovdiv.BGR/03.10/3[D4]	Acute measles, vaccination failure
13	35 y	Unknown	2010 Jun 2/Jun 7	Pos	Pos	Low (27%)	U pos, TS pos	MVs/Plovdiv.BGR/23.10/1[D4]	Acute measles
14	19 y	MMR1, 1991/ MMR2, 2002	2010 Jun 3/Jun 7	Equivocal	Pos	Equivocal (46%)	U pos, TS pos	MVs/Plovdiv.BGR/23.10/2[D4]	Secondary vaccination failure?
15	11 y	MMR1, 2001/ MMR2 2010 May 27	2010 Jun 2/Jun 7	Pos	Neg	ND	U pos, TS pos	MVs/Plovdiv.BGR/23.10/3[D4]	Acute measles, vaccination failure
16	9 y	Unknown	2010 Jun 4/Jun 7	Pos	Neg	ND	U neg, TS pos	MVs/Plovdiv.BGR/23.10/4[D4]	Acute measles
17	14 y	MMR1, 1998	2010 Jun 4/Jun 7	Pos	Neg	ND	U pos, TS pos	MVs/Plovdiv.BGR/23.10/5[D4]	Acute measles, vaccination failure
18	6 y	MMR1, 2004	2010 Jun 3/Jun 7	Pos	Neg	ND	U pos, TS pos	MVs/Plovdiv.BGR/23.10/6[D4]	Acute measles, vaccination failure
19	22 y	Unknown	2011 Mar 6/Mar 11	Pos	Pos	93%	U pos	MVs/VelikoTarnovo.BGR/10.11/1[D4]	Secondary vaccination failure
20	32 y	Unknown	2011 Mar 8/Mar 11	Pos	Pos	86%	U pos	MVs/VelikoTarnovo.BGR/10.11/2[D4]	Secondary vaccination failure

*Ig, immunoglobulin; MMR, measles, mumps, rubella vaccine; MMR1, 1 dose of MMR vaccine; MMR2, two doses of MMR vaccine; pos, positive; ND, not determined; U, urine; neg, negative;–, not investigated (serum sample not available); TS, throat swab. Patients were hospitalized in distinct districts of Bulgaria; specimens were investigated in the World Health Organization Regional Reference Laboratory, Berlin, Germany.
